# Ultrafast evolution and transient phases of a prototype out-of-equilibrium Mott-Hubbard material

**DOI:** 10.1038/s41467-019-11743-3

**Published:** 2019-09-06

**Authors:** David Moreno-Mencía, Alberto Ramos-Álvarez, Luciana Vidas, Seyed M. Koohpayeh, Simon Wall

**Affiliations:** 1 ICFO-Institut de Ciències Fotòniques, The Barcelona Institute of Science and Technology, 08860 Castelldefels, Barcelona Spain; 2 Department of Physics and Astronomy, Institute for Quantum Matter, Johns Hopkins University, Baltimore, MD 21218 USA

**Keywords:** Electronic properties and materials, Phase transitions and critical phenomena

**Arising from** G. Lantz et al. *Nature Communications* 10.1038/ncomms13917 (2017).

Lantz et al^1^. report a transient non-thermal phase in V_2_O_3_ following photoexcitation with femtosecond pulses of light at 800 nm arising from both the paramagnetic insulating (PI) and paramagnetic metallic (PM) phases of V_2_O_3_. The transient state is said to be stabilised by lattice distortion due to an overpopulation of the a_1g_ electronic orbital. This is observed through a hardening of the A_1g_ coherent phonon mode by 14% from the value observed in Raman measurements at equilibrium. We have repeated the optical measurements, performed on the paramagnetic metallic phase, on several different samples and found no evidence for light-induced phonon hardening. Raman and time-domain signals are found to be in good agreement. Instead, we find that the equilibrium A_1g_ mode frequency is sample dependent, with values spanning 0.6 THz in the three samples measured. Our results show that V_2_O_3_ does not undergo any anomalous photo-induced phase transition and that the excited state is most likely a thermal one.

The data shown in Fig. 1 comes from a single crystal of the PM phase of V_2_O_3_. The samples were grinded and polished using grinding disk papers and polishing cloths with diamond suspension down to 3 µm to give a (001) surface normal. The room temperature lattice parameters of a = 4.9535(5) Å and c = 14.0043(4) Å were measured. A sharp first-order insulator–metal phase transition at 165 K, often resulting in the crystal shattering, shows that our samples are of high quality and correspond to stoichiometric V_2_O_3_.

**Fig. 1:**
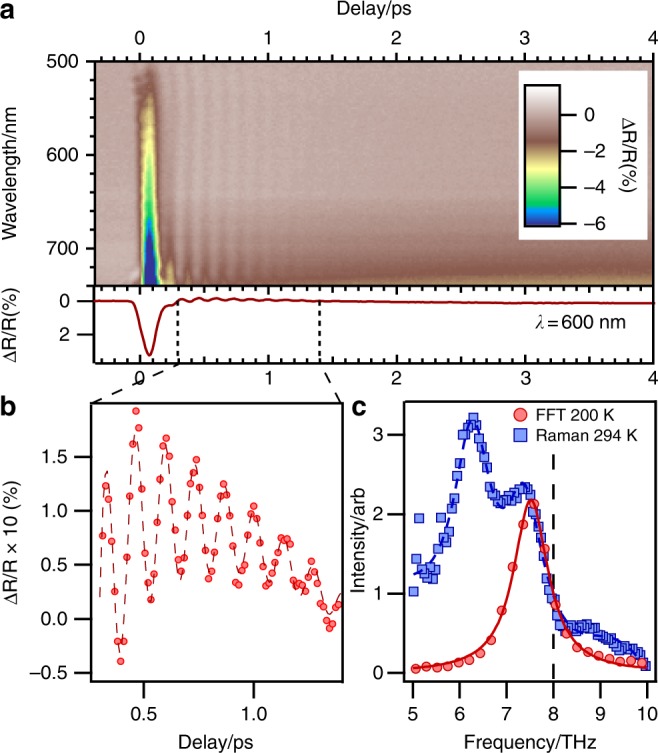
Transient response of V_2_O_3_ to photoexcitation. **a** Time- and wavelength-resolved response of V_2_O_3_ after excitation with a 40 fs 1.5 eV pump pulse at 1 kHz repetition rate, a pump fluence of 8 mJ cm^−2^ and a base temperature of 200 K. The 2D data are averaged over a 100-nm-wide region, centred at 600 nm to extract a 1D time trace. **b** Zoom of the data to show the measured coherent phonon oscillations (circles) together with the fit (dashed line) with a 7.4 THz mode. **c** Fourier transform of the same data (blue circles) after differentiation, in order to remove the slow dynamics. A Lorentzian fit (blue line) gives a central frequency of 7.5 THz. The extracted frequency is in good agreement with the Raman data, taken at room temperature (blue line). The first peak (~6 THz) corresponds to the E_g_ mode, which is not measured in our time-resolved measurement, and the second corresponds to the observed A_1g_ mode. The mode frequencies are in good agreement with Ref. ^3^. The black dashed line is the blue-shifted frequency reported in Ref. ^1^

Experiments were carried out in an optical cryostat held at 200 K. In order to reproduce the experimental conditions reported in Ref. ^1^, the pump–probe measurements were performed in a cross-polarised geometry at near normal incidence, and the pump fluence was set to 8 mJ cm^−2^. One significant difference in our measurement is that we use a broadband optical probe and frequency-resolve detection, which provide greater sensitivity to the phonon oscillation. Figure 1a shows the wavelength dependence of the transient optical response after correcting for the chirp of the white light probe. The response is similar to that observed by Ref. ^1^ with a spike-like feature near *t* *=* 0 and coherent oscillations over a slowly varying background. The response is similar at all wavelengths, but the relative strength of the peak and oscillation amplitude changes for different wavelengths. In order to obtain the phonon frequency, we averaged a 100-nm section of the wavelength data, centred at 600 nm, to produce a single time trace. Figure 1b shows a zoom of the transient, together with a time dependent fit to the transient data, which resulted in a central frequency of 7.41 ± 0.01 THz (247 cm^−1^). In addition, we remove the background by differentiating the transient response, as performed by Ref. ^2^, which acts as a complex low-pass filter for the slow dynamics. A fast Fourier transform (FFT) of the result was then calculated, after excluding the range close to the spike at t = 0. The resulting FFT is shown in Fig. 1c. A Lorentzian fit to the FFT gives a central frequency of 7.51 ± 0.01 THz (250 cm^−1^). The discrepancy in frequency obtained from the two methods of analysis of the same data is larger than the error reported by the fitting algorithm in either case. This shows that the main error in determining the mode frequency arises from how the background is processed. We determine the frequency of the mode to be 7.46 ± 0.05 THz by using the mean and standard deviation of the two fit results. This frequency is significantly slower than 8 THz, the response observed in Ref. ^1^.

Raman measurements were also performed on the same sample at room temperature. Here, a small red shift can be expected due to the thermal induced softening of the mode, i.e., we expect it to be slightly lower in frequency than the data recorded at 200 K. The data were recorded in an unpolarised backscattering geometry with a 785 -nm laser, and the results are also plotted in Fig. 1c. The Raman measurements are in good agreement with previous reports in the literature^3,4^, again attesting to the good quality of our crystals. We used a multi-Lorentzian fit and linear background to extract the central frequency of the Raman mode and a value of 7.39 ± 0.03 THz. Again, this value can change if the background function is also modified, and the true error will be larger than the value reported by the fit. However, even with this lower bound, the Raman and time-domain frequencies obtained can be considered the same, to within the combined error bars. Therefore, we conclude that light does not induce a significant blue shift.

In order to investigate the discrepancy further, we performed a range of fluence-dependent measurements on different samples of stoichiometric V_2_O_3_ and at different fluences. Samples 1 and 2 were grown and polished by our collaboration at John Hopkins University using the methods described above. The data from other samples were acquired at facilities in our lab at ICFO. We show these data only for the purpose of showcasing range of frequencies that can be observed from sample to sample, but not for the purpose of reporting scientific values. Figure 2 plots the mode frequency as a function of power resulting from a Lorentzian fit to the Fourier transform of the background subtracted data. In sample 1, we find that the frequency of the response can change by 3% by moving to different spots in the same sample, which otherwise showed no obvious difference in the optical appearance (sample 1a vs sample 1b in Fig. 2), indicating that residual strain from polishing may change the frequency. Agreement can also be found between different samples (samples 1a and 2). The largest discrepancy we have observed is 0.6 THz (sample 1b and sample 3). This shows the measured mode frequency is very sample dependent and depends strongly on sample preparation.

**Fig. 2:**
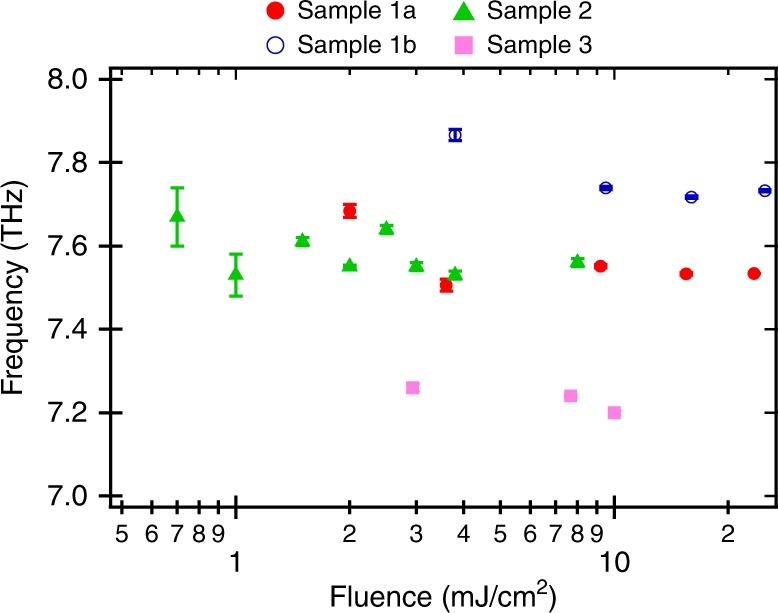
Fluence dependence of the time-domain frequency for several samples of un-doped V_2_O_3_. All measurements were performed at room temperature. Samples 1a and b where measurements performed at two points on the same sample. A variation of 0.6 THz (~8%) is found over the samples measured. Error bars correspond to the error from the fit of the Lorentzian obtained from Fourier transforming the time-domain data and are an underestimate. Source data are provided as a Source Data file

Lantz et al.^1^ argue that the blue shift is driven by the population in the a_1g_ electronic level. Therefore, one would expect the magnitude of the shift to increase or decrease with increasing or decreasing fluence as more or fewer electrons are excited into that orbital. However, we only observe a small red shift with fluence, despite changing the fluence by almost two orders of magnitude. Over this range, the phonon amplitude and reflectivity change continue to increase, showing that the a_1g_ orbital population has not saturated.

In conclusions, we find no evidence for a transient non-thermal phase in V_2_O_3_ that is stabled by a lattice distortion and photo-induced hardening of the A_1g_ phonon mode as claimed in Ref. ^1^. Good agreement between Raman and time-domain signals can be found when the measurements are performed on the same sample. There is no light-induced hardening and we do not find any evidence for control of the lattice structure by selective excitation of the a_1g_ orbital in this compound. Our results show that there can be a large variation in A_1g_ phonon mode frequency both within and between samples, which needs to be taken into account when comparing measurements with literature values.

The data that support the findings of this study are available from the corresponding author upon reasonable request.

## Supplementary information

Source Data
